# The role of negative pressure wound therapy with instillation and dwell time in the treatment of deep sternal wound infections—A retrospective cohort study

**DOI:** 10.1002/hsr2.1430

**Published:** 2023-07-16

**Authors:** Olimpiu Bota, Feras Taqatqeh, Florian Bönke, Jörg Nowotny, Klaus Matschke, Kevin Bienger, Adrian Dragu

**Affiliations:** ^1^ Faculty of Medicine Carl Gustav Carus University Center for Orthopedics, Trauma and Plastic Surgery Dresden Germany; ^2^ Department of Cardiac Surgery University Heart Center Dresden Dresden Germany

**Keywords:** cardiac surgery, instillation, negative pressure wound therapy, surgical wound infection, wound healing

## Abstract

**Background and Aims:**

Negative pressure wound therapy (NPWT) has gained a central role in the treatment of deep sternal wound infections (DSWIs) after median thoracotomy. Our study aims at proving the safety of using NPWT with instillation and dwell time (NPWTi‐d) in the treatment of DSWI.

**Methods:**

We retrospectively evaluated the patients who were treated at our institution between March 2018 and November 2021 for DSWI after radical sternectomy using NPWT or NPWTi‐d. The NPWTi‐d was applied to start the first postoperative day using 75 mmHg negative pressure for 3 h, followed by instillation of sodium hypochlorite <0.08% with a 3‐min dwell time.

**Results:**

The NPWTi‐d group showed a shorter length of stay (29.39 ± 12.09 vs. 39.54 ± 17.07 days; *p* = 0.049), a shorter elapsed time between the debridement and the flap coverage (7.18 ± 4.27 vs. 11.86 ± 7.7 days; *p* = 0.003) and less operative or nonoperative dressing changes (1.73 ± 1.14 vs. 2.68 ± 56; *p* < 0.001). The in‐hospital mortality was 8.2%, with no significant differences between the two groups (*p* = 1).

**Conclusion:**

NPWTi‐d can be safely employed in the treatment of DSWI. Further prospective randomized studies need to establish the role of NPWTi‐d in the control of infection and biofilm as well as in wound healing.

## Key points

Negative pressure wound therapy with instillation and dwell time is a safe and helpful tool in the treatment of deep sternal wound infections. Our retrospective cohort analysis showed in 87 patients that the use of instillation and dwell time results in fewer dressing changes, faster flap coverage, and fewer hospitalization days with the same complication rate compared to the classical NPWT therapy.

## 1 INTRODUCTION

Deep sternal wound infection (DSWI) represents a serious complication of median thoracotomy for cardiac surgery, with an average incidence of 1.6%.^1^ Patients who develop DSWI have suffered an open cardiac intervention and most frequently have several comorbidities like diabetes mellitus, obesity, chronic obstructive pulmonary disease (COPD), or peripheral arterial disease which all complicate the treatment and increase mortality.^2^

The evolution of negative pressure wound therapy (NPWT) has radically changed the treatment course and outcomes of patients with DSWI, decreasing the in‐hospital mortality from 36% to 52%^3^ before the NPWT era to around 10% nowadays.^4^ NPWT ensures the drainage of secretions, promotes vascularity and granulation while sealing the wound. It can be used either as a bridge until flap closure or as vacuum‐assisted wound closure.^5^, ^6^

The further development of NPWT has led to the addition of instillation and dwell time (NWPTi‐d). Since its commercial introduction in 2003, NPWTi‐d has found a wide range of applications, including pressure sores, burns, traumatic wounds, diabetic and venous ulcers, or open amputations. The evidence shows that in these types of wounds NPWTi‐d causes a reduction in hospital stay, time to wound closure, number of surgical debridements and dressing changes.^7^ Furthermore, NPWTi‐d has been proven to accelerate the granulation by up to 43% and reduce the bioburden by up to 48%.^8^ The technique involves the instillation of a solution with a recommended dwell time of 10–20 min and a negative pressure time of 2–4 h at −125 mmHg. There is no consensus concerning the instillation solutions, which can be saline, hypochlorite, biguanide, or iodine solutions.^9^

Although the features of NPWTi‐d could accelerate infection control in DSWI, there is little data in the literature about its usage in the treatment of DSWI and the present guidelines for NPWTi‐d do not mention these wounds.^9^, ^10^ Our study aimed to evaluate the safety of NPWTi‐d in the treatment of DSWI. Secondarily, we aimed to identify the possible benefits of NPWTi‐d as opposed to NPWT in treating these patients.

## 2 METHODS

We retrospectively examined all patients who were treated for DSWI at our institution between March 2018 and November 2021. The inclusion criteria were the presence of DSWI with an open thorax and treatment with either NPWT or NPWTi‐d. The exclusion criterion was the presence of DSWI without an open thorax. We identified 85 patients to be included in the study, out of which 48 were in the NPWTi‐d group (A) and 37 were in the NPWT group (B). The data were retrospectively gathered in Excel 365 (Microsoft Corporation). The statistical analysis was performed using JASP version 0.16.4 (University of Amsterdam); for nominal, the Fisher's exact test was performed. The continuous data were tested for normal distribution using the Shapiro–Wilk test. The one‐sided *t* test was used with normal distributions and the one‐sided Mann–Whitney *U* test (MWU) was used in the other cases. Statistical significance was assumed when *p* < 0.05.

The study was approved by the institutional review board (EK 387082020).

### 2.1 Therapeutical procedure

**Figure 1:**
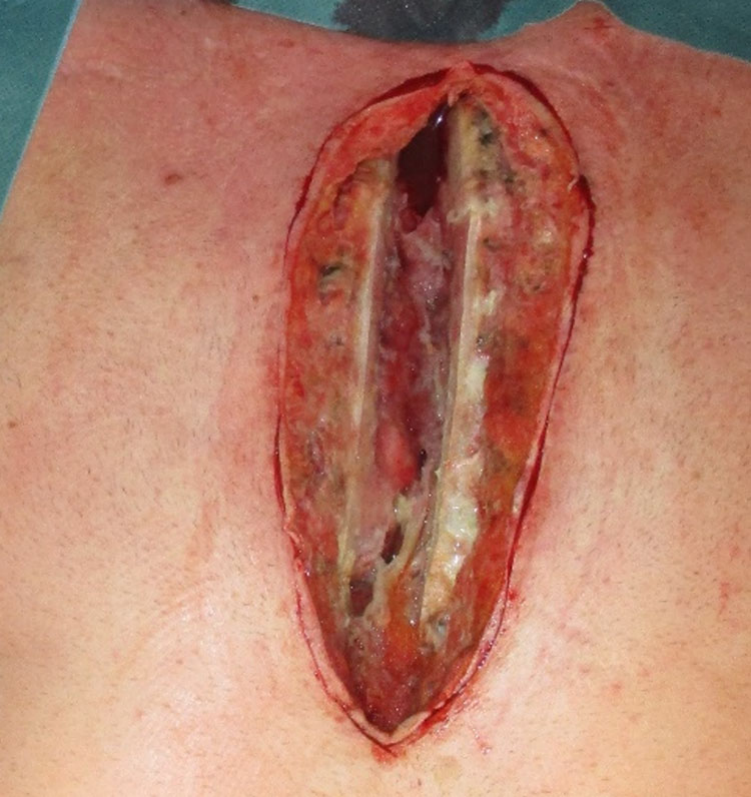
DSWI with open thorax before debridement.

**Figure 2:**
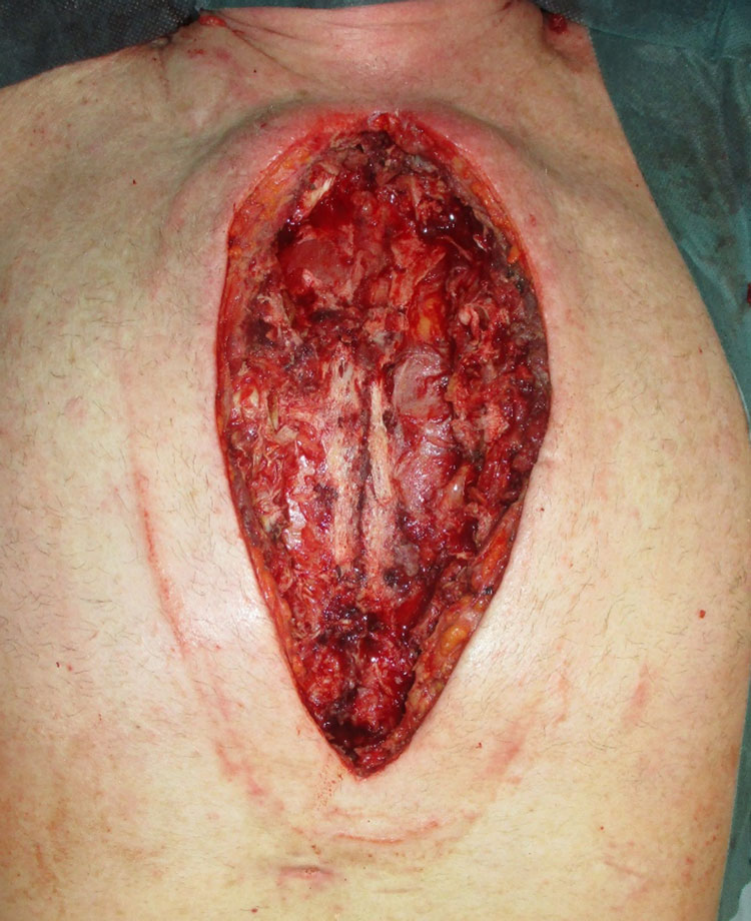
Mediastinal wound after radical en bloc sternectomy.

**Figure 3:**
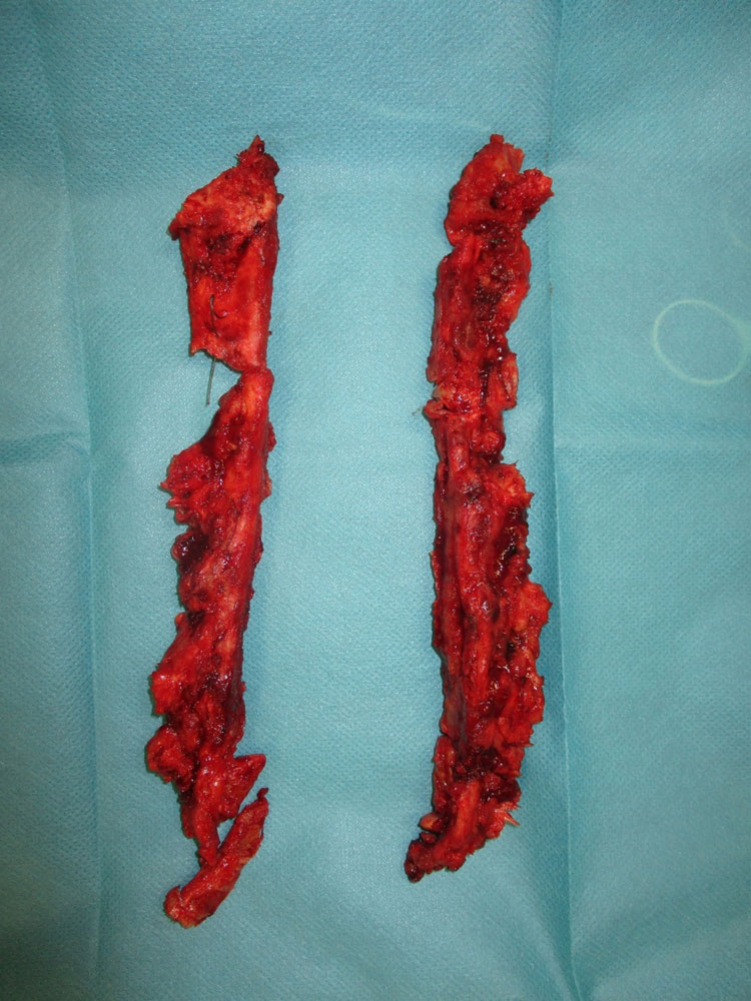
Resected hemisternum specimens.

After the diagnosis of DSWI (Figure 1), a radical sternal debridement is performed with radical sternectomy (Figures 2 and 3). If the pleura parietalis is opened during the debridement, the opening is closed either by suture or by a fibrin sealant patch. If, due to adhesions, the lung parenchyma is opened, a similar procedure is performed. In cases where the pericardium is opened after the debridement, closure is tempted using a direct suture. If this is not possible, NPWTi‐d is contraindicated due to the theoretical risk of pericardial tamponade.

Finally, if bleeding control is achieved, foam is used to obliterate the mediastinal wound, and then the adhesive drape is used to seal the wound. A trackpad with two tubes is applied. One tube is connected to the canister tubing and the NPWT device is set to 75 mmHg. A higher pressure has been proven in our experience to increase the pain and discomfort of the patient. The second tube is firstly clamped and will be later used for instillation. To avoid washing out the clots and causing postoperative bleeding, the NPWTi‐d is started on the first postoperative day. At this moment, the second tube is declamped and connected to the instillation device, using a sodium hypochlorite solution <0.08%. The instilled quantity is estimated at the first application so that the solution will not spill out of the wound and detach the adhesive drape. Usually, 30–50 mL is necessary for the mediastinal wound. The negative pressure therapy time is set for three hours, with a dwell time of three minutes. This ensures that antimicrobial action takes place while minimizing the risk of drape detachment with loss of vacuum effect.

**Figure 4:**
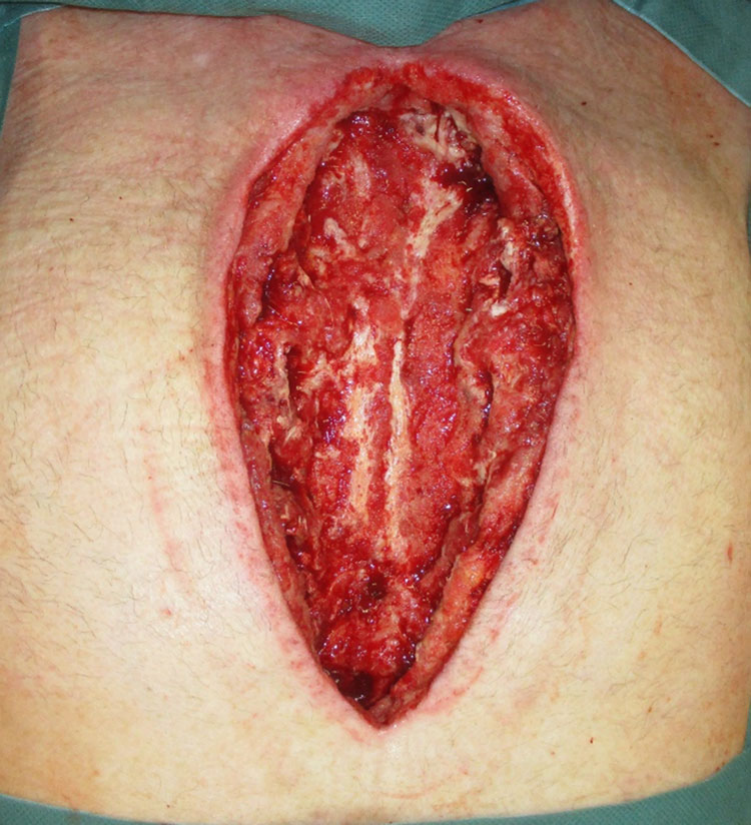
Mediastinal wound after 5 days NPWTi‐d.

**Figure 5:**
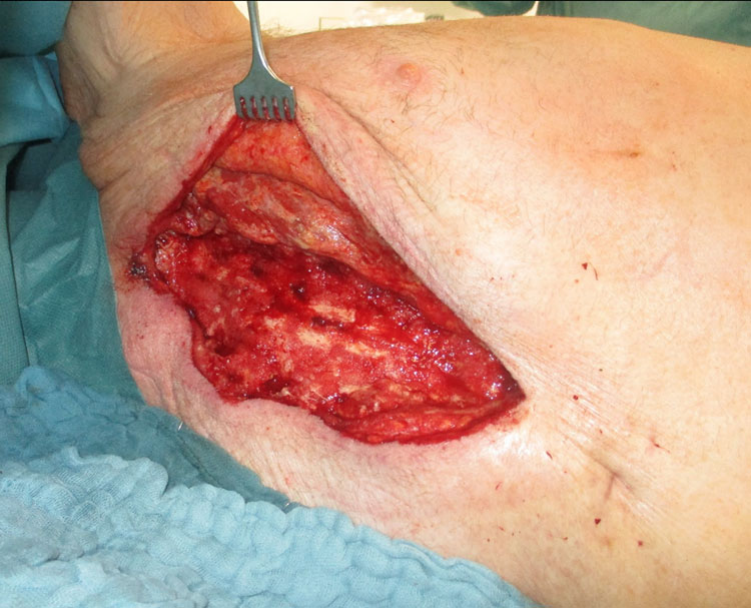
Mediastinal wound after 10 days NPWTi‐d. Patient in lateral decubitus for flap closure.

**Figure 6:**
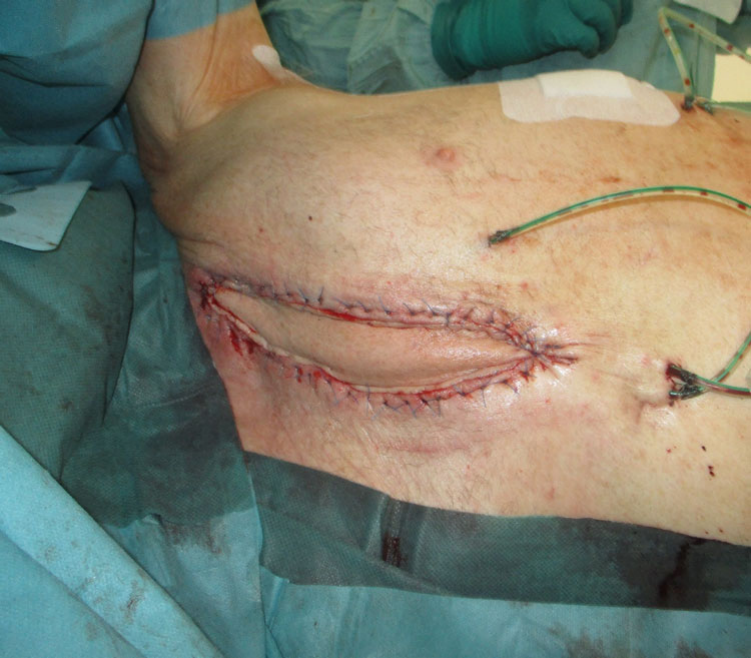
After closure with pedicled Latissimus dorsi musculocutaneous flap.

**Figure 7:**
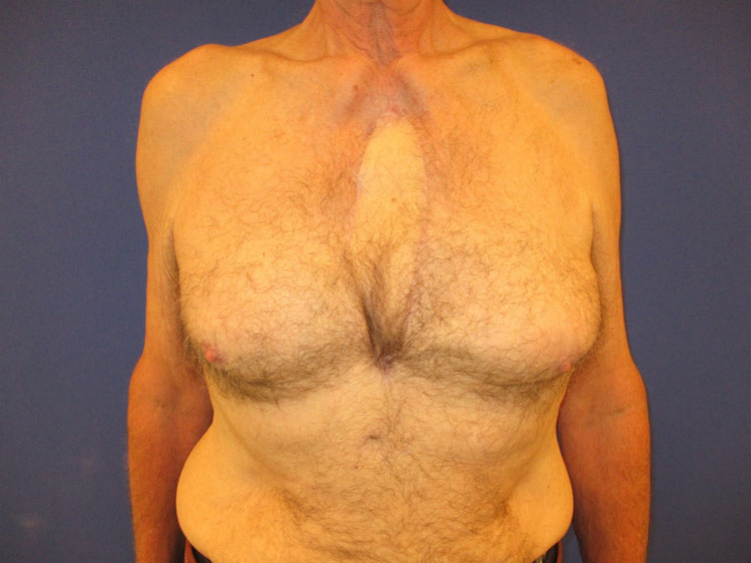
Result 1‐year postoperative.

If, due to anticoagulation and anti‐aggregation, there is diffuse bleeding after the debridement, which cannot be controlled, the wound is packed with cotton gauze and sealed with adhesive tape. The NPWT dressing is then applied on the first postoperative day and the instillation is started. The flap closure is usually planned in the subsequent 3–5 days after debridement (Figures 4 and 5). The condition for flap closure is a viable wound without necrotic tissue and without visible purulent secretions. As flap closure, we standardly use the pedicled latissimus dorsi musculocutaneous flap as described by Bota et al. ^11^ (Figures 6 and 7) or alternatively the pectoralis major muscle flap, either unilateral or bilateral. If the general condition of the patient does not allow the flap procedure, the dressing change needs to take place within 4–5 days or whenever the vacuum seal of the wound is lost.

## 3 RESULTS

We identified 95 patients with DSWI. Eight patients had only a partial sternal infection without an open thorax. Two patients died before the NPWT treatment could be initiated. Eighty‐five patients could be included in the study, 19 females and 66 males, with a mean age of 67.37 (45‐85) years. Forty‐eight patients were treated with NPWTi‐d and 37 were treated with NPWT. There were no significant differences between the two groups concerning age, body mass index (Student *t* test), sex, smoking, alcohol consumption, American Society of Anesthesiologists classification, congestive heart failure, myocardial infarction, arterial hypertension, arrhythmia, endocarditis, type of heart surgery (coronary bypass, valve replacement, aortic prosthesis), COPD, renal insufficiency or stroke (Fischer's exact test [FET]). There were significantly more patients with diabetes mellitus in the NPWTi‐d group (35 vs. 17, *p* = 0.014, FET), although the insulin intake was similar between the groups (*p* = 0.151, FET; Table 1).

**Table 1:** Patient characteristics and comorbidities.

	NPWT‐id	NPWT	Total	*p* value
Age	67.438	67.297	66.376	0.534
Female	11	8	19	1
Male	37	29	66	
BMI	29.602	29.265	29.456	0.614
ASA	3.167	3.189	3.176	0.783
Smoking	19	14	33	1
Alcohol consumption	6	5	11	1
Diabetes mellitus	35	17	52	0.014
Insulin intake	16	7	23	0.151
Congestive heart failure	41	30	71	0.769
Myocardial infarction	15	14	29	0.645
Arterial hypertension	45	33	78	0.694
Arrhythmia	23	20	43	0.663
Endocarditis	4	3	7	1
Valve replacement	31	15	46	0.079
Aortic prosthesis	5	3	8	1
Coronary bypass	40	29	69	0.567
Average number of bypasses	1.854	1.541	1.718	0.469
COPD	7	7	14	0.769
Renal insufficiency	23	15	38	0.518
Deceased	4	3	7	1

Abbreviations: ASA, American Society of Anesthesiologists; BMI, body mass index; COPD, chronic obstructive pulmonary disease.

When evaluating the intraoperative incidents, we found no significant difference between the two groups considering the opening of the parietal pleura, the lung parenchyma, or the pericardium (Table 2).

**Table 2:** Frequency of intraoperative incidents.

	NPWTi‐d		NPWT		*p* value (FET)
	No	Yes	No	Yes	
Opening pleura parietalis	35	13	26	11	0.81
Opening lung parenchyma	36	12	27	10	1
Opening pericardium	46	2	32	5	0.23

Abbreviation: FET, Fischer's exact test.

When evaluating the length of hospital stay, the patients with NPWTi‐d (29.39 ± 12.09 days) were discharged significantly earlier than the patients without NPWT (39.54 ± 17.07; *p* = 0.049, MWU). The time elapsed between the debridement and the flap coverage was significantly shorter in the NPWTi‐d group (7.18 ± 4.27 days) than in the NPWT group (11.86 ± 7.7 days; *p* = 0.003, MWU). Furthermore, the number of surgeries or bedside dressing changes performed until flap closure was significantly less in the NPWTi‐d group (1.73 ± 1.14 vs. 2.68 ± 1.56; *p* < 0.001, MWU; Table 3).

**Table 3:** Treatment outcomes.

	NPWT‐id	NPWT	Total	*p* value
Length of hosptital stay	29.39 ± 12.09	39.54 ± 17.07	32.07 ± 15.02	0.049
Time elapsed between debridement and flap coverage	7.18 ± 4.27	11.86 ± 7.7	9.22 ± 6.4	0.003
Number of dressing changes	1.73 ± 1.14	2.68 ± 1.56	2.14 ± 1.41	<0.001

The CT scans before the debridement showed no pneumothorax, whereas after debridement in three cases and after flap coverage in five cases an asymptomatic pneumothorax was diagnosed, without a significant difference between the two examined groups (*p* = 0.58 and p = 1, FET). No pericardial tamponade was recorded. Moreover, the thorax CT scans were evaluated for the occurrence of pleural effusion before and after the debridement as well as after the flap coverage. The findings showed no significant differences between the two examined groups in any of the examined moments (Table 4).

**Table 4:** Frequencies of pleural effusion and pneumonia. Fischer's exact test was applied for NPWTi‐d and NPWT‐groups.

Complication	Point of time	NPWTi‐d		NPWT		*p* value
		No	Yes	No	Yes	
Pleural effusion left	Before debridement	42	6	33	4	1
	After debridement	34	14	37	10	1
	After flap closure	40	8	28	9	0.42
Pleural effusion right	Before debridement	44	4	34	3	1
	After debridement	35	13	26	11	0.81
	After flap closure	39	9	29	9	0.53
Pneumonia	Before debridement	48	0	37	0	1
	After debridement	44	4	35	2	0.69
	After flap clousre	44	4	33	4	0.72

Finally, the in‐hospital mortality was 8.2% (seven patients), with no significant differences between the two groups (4 vs. 3 patients, *p* = 1, FET).

## 4 DISCUSSION

In the present study, we compared the complications and early outcomes of NPWTi‐d and NPWT in treating the DSWI and found no significant difference in the rate of perioperative complications, with low overall mortality. Moreover, NPWTi‐d showed a trend toward fewer dressing changes, faster flap coverage, and shorter length of hospital stay.

The introduction of NPWT has revolutionized the treatment of DSWI. The high mortality of this dreadful complication could be reduced from around 50% before the NPWT down to around 10% nowadays.^3^, ^4^ The impact on NPWT can be explained on one hand through the drainage of secretions, promotion of vascularity and granulation and on the other hand by the mechanical stabilization of the open thorax and sealing of the open mediastinal wound. The procedure has also greatly reduced the need for bedside dressing changes, which classically lead to pain, further wound contamination, and possible major organ injury. Although some authors propose to use of NPWT as a means of wound closure,^6^ we believe that the swift and effective flap closure ensures better results with less stress to the patient and better survival rates.^5^

One major risk to the employment of NPWT in DSWI is the risk of organ rupture by adhesion and ingrowth of the tissue into the foam. While the lungs can be relatively easily resealed as described before and the great vessels are seldomly directly exposed, there are several reports of bypass vessel or right ventricular rupture with NPWT dressing change with catastrophic consequences.^12^, ^13^ This complication may occur when the pericardium has been partially removed and the myocardium is directly exposed. In these cases, we employ for protection a perforated silicone sheath between the exposed organ and the foam.

The development of NPWTi‐d has further improved the beneficial impact of NWPT by enhancing the granulation and reducing the bioburden.^8^, ^14^ While the control of the infection and wound stabilization are prerequisites for successful wound closure in DSWI, NPWTi‐d has not been yet largely deployed in these cases. Concerns of organ rupture or leakage of instillation solution in the pleural cavities or the lungs have prevented so far the broad use of NPWTi‐d in DSWI.

For the utilization of NPWTi‐d, guidelines have been issued, which recommend its use in different types of chronic wounds, including the use with caution in wounds that contain appropriately protected organs or vessels.^15^ Although the DSWI were not specifically described so far in these guidelines, one could allocate these wounds in the latter description.

The recommended negative pressure time is 2–3 h, with a dwell time of 10 min.^15^ In our opinion, the time needed for the instillation solution to have an effect should not interfere with the tightness of the dressing. Especially in a mobile patient, if the instillation time occurs for prolonged periods and too often, the fluid may detach the adhesive drape and require an immediate dressing change, which in DSWI represents an additional burden for the patient and the hospital. Several instillation solutions have been recommended so far, including normal saline, acetic acid solutions (0.25%–1%), polyhexamethylene biguanide (PHMB) (0.1%) with betaine (0.1%), hypochlorous acid solutions, and sodium hypochlorite solutions (0.125%).^15^ In our practice, we use a solution of sodium hypochlorite/hypochlorous acid <0.08% (NaOCl/HOCl). The NaOCl has been proven in vitro to have efficiency against the biofilm formed by *Staphylococcus aureus* (SA), *Staphylococcus epidermidis* (SE), *Pseudomonas aeruginosa* (PA) and other wound pathogens, which are commonly encountered in DSWI.^2^, ^16^, ^17^ As described in 1915 by Henry Drysdale Dakin, the Dakin solution classically contains 0.5% buffered sodium hypochlorite.^18^ As the active ingredients in this solution can have a toxic effect on different cells like fibroblasts, macrophages, or stratum corneum, lower doses of this antiseptic solution need to be used in open wounds, as the toxicity appears to be dose‐related.^19^ On one hand, the treatment with NaOCl achieves in vitro complete growth inhibition of SA and SE after 2 min and of PA after 5 min ^17^ and on the other hand, NaOCl is inactivated when coming in contact with proteins and blood from the wound.^20^ Considering all these, we find that instillation with NaOCl with a low concentration (0.08%) for 3 min every 3 h may provide an optimal antimicrobial effect while minimizing the toxicity and the risk of seal compromise. Nevertheless, a 5‐min dwell time can be taken into consideration, to maximize the antimicrobial effect. The recommended negative pressure of −125 mmHg causes in our experience pain and discomfort, and therefore, we employ 75 mmHg, which is better tolerated by the patients.

The first five cases of DSWI treated with instillation were reported in 1969 by Bryant et al., who used plastic tubes to irrigate the mediastinal wounds with antibiotic solutions over a prolonged period.^21^ Although the first description of an NPWT system with instillation was in 1998^22^ and the commercial introduction occurred in 2003,^7^ there are so far few reports about the usage of NPWTi‐d to treat DSWI. Schreiner et al. published a series of 11 cases, out of which 3 cases of DSWI, 6 cases of pleural empyema, and 2 cases of sternoclavicular joint infection.^23^ The authors used instillation with PHMB 0.2% for 18 min followed by lavage with Ringer solution and a 2‐h negative pressure phase and reported no adverse effects and wound closure in all patients. Particular to this study is the successful intrathoracic application of NPWTi‐d with PHMB, which compared to NaOCl has a longer bactericide effective time and also may negatively affect endothelial cells and osteoblasts at therapeutic concentrations.^20^, ^24^ Another case report presents the treatment of a DSWI with *Mycoplasma hominis* using NPWTi‐d for 26 days using PHMB 0.02% with 10 min dwell time and 3 h negative pressure at 125 mmHg. Finally, the sternum could be rewired and the wound secondarily closed.^25^ Except for the different NPWTi‐d treatment regime, this study had no sternectomy, compared to our work.

Chowdhry et al. retrospectively reported on 30 patients with DSWI, out of which 15 were treated with NPWTi‐d and 15 with conventional wet‐to‐moist dressings.^10^ They used 1/8 Dakin solution (0.0625% buffered NaOCl) with a 20‐min dwell time and 2‐h negative pressure therapy. Additionally, the first group received postoperatively incisional NPWT. They could prove a significantly shorter time to closure, fewer dressing changes, and shorter drain duration in the NPWTi‐d group. Compared to our work, this study didn't perform a radical sternectomy; wound closure was performed using bilateral pectoralis major muscle flaps. More importantly, for the control group, conventional dressings were used, which expectedly would take longer to control the infection. In this study, reticulated open‐cell foams with NPWTi‐d were used. These foams help achieve better local debridement while reducing the thick exudate and heavy bioburden. Due to the risk of tissue in‐growth into the foam cells and the risk of injuring the mediastinal organs, we currently do not apply this dressing in DSWI.

Our study suggests that NPWTi‐d can be safely applied in DSWI without an increased risk of mediastinal or thoracic complications. While the two groups were similar in terms of patient characteristics, comorbidities, the intraoperative opening of the pleura parietalis, lung parenchyma, and pericardium, there were similar rates of postoperative pneumothorax, pleural effusion, and pneumonia, which could have been caused by the instillation solution. Even more important, there were no serious adverse effects like pericardial tamponade or toxic reactions to NaOCl. The cohort showed low in‐hospital mortality as compared to the literature^4^, ^11^ and no differences between the two examined groups.

Although the goal of our study was to demonstrate the safety of NPWTi‐d in the treatment of DSWI, we could additionally show a tendency toward fewer dressing changes, faster flap coverage, and fewer hospitalization days. This could be attributed to the faster and more efficient stabilization of the mediastinal infection, as all 85 patients received a similar standardized radical debridement with sternectomy. Our study was nevertheless retrospective and a future prospective randomized controlled study should compare NPWTi‐d with NPWT in terms of reducing the bioburden and biofilm.

The main limitation of this study is its retrospective character, which precluded proper randomization of the two study groups. The number of patients in the two groups was nevertheless significant for a rare disease like DSWI. The cautious deployment of both negative pressure therapy techniques ensured

NPWTi‐d can be safely employed in the treatment of DSWI with an improved length of hospital stay, less time elapsed from debridement until flap closure, and fewer dressing changes. Further prospective randomized studies need to establish the role of NPWTi‐d in the control of infection and biofilm as well as in wound healing.

## AUTHOR CONTRIBUTIONS

**Olimpiu Bota**: Conceptualization; data curation; investigation; methodology; project administration; supervision; validation; writing—original draft; writing—review and editing. **Feras Taqatqeh**: Validation; writing—review and editing. **Florian Bönke**: Validation; writing—review and editing. **Jörg Nowotny**: Validation; writing—review and editing. **Klaus Matschke**: Project administration; resources; supervision; writing—review and editing. **Kevin Bienger**: Data curation; investigation; methodology; writing—review and editing. **Adrian Dragu**: Resources; supervision; validation; writing—review and editing.

## CONFLICT OF INTEREST STATEMENT

The authors declare no conflict of interest.

## ETHICS STATEMENT

Institutional review board (IRB) approval from the Ethics Committee of TU Dresden was obtained (BO‐EK‐387082020). An informed consent to participate was waived by the Ethics Committee of TU Dresden. All methods were carried out in accordance with the guidelines and regulations.

## TRANSPARENCY STATEMENT

The lead author Olimpiu Bota affirms that this manuscript is an honest, accurate, and transparent account of the study being reported; that no important aspects of the study have been omitted; and that any discrepancies from the study as planned (and, if relevant, registered) have been explained.

## Data Availability

The data sets used and/or analyzed during the current study are available from the corresponding author upon reasonable request.
